# DoPI: The Database of Pollinator Interactions

**DOI:** 10.1002/ecy.3801

**Published:** 2022-08-29

**Authors:** Nicholas J. Balfour, Maria Clara Castellanos, Dave Goulson, Andrew Philippides, Chris Johnson

**Affiliations:** ^1^ School of Life Sciences University of Sussex Brighton UK; ^2^ Department of Engineering and Informatics University of Sussex Brighton UK

**Keywords:** bipartite networks, conservation, database, flower visitors, interactions, phenology, plants, pollination, pollinators

## Abstract

Despite the importance of pollinating insects to natural environments and agriculture, there have been few attempts to unite the existing plant–pollinator interaction datasets into a single depository using a common format. Accordingly, we have created one of the world's first online, open‐access, and searchable pollinator–plant interaction databases. DoPI (The Database of Pollinator Interactions) was built from a systematic review of the scientific literature and unpublished datasets requested from researchers and organizations. We collated records of interactions between British plant and insect flower–visitor species (or genera), together with associated metadata (date, location, habitat, source publication) when available. The dataset currently (December 2021) contains 101,539 records, detailing over 320,000 interactions. The number of interactions (i.e., the number of times a pairwise species interaction was recorded per occasion) varies considerably among records, averaging 3.6. These include records from 1888 pollinator species and 1241 plant species, totaling >17,000 pairwise species interactions. By combining a large volume of information in a single repository, DoPI can be used to answer fundamental ecological questions on the dynamics of pollination interactions in space and time, as well as applied questions in conservation practice. We hope this dynamic database will be a useful tool not only for researchers, but also for conservationists, funding agencies, governmental departments, beekeepers, agronomists, and gardeners. We request that this paper is cited when using the data in publications and individual studies when appropriate. Researchers and organizations are encouraged to add further data in the future. The database can be accessed at: https://www.dopi.org.uk/.

## CONFLICT OF INTEREST

The authors declare no conflict of interest.

## Supporting information


Data S1

## Data Availability

The complete data set for this release is available as Supporting Information. The updated database can be accessed at: https://www.dopi.org.uk/.

