# Supplementary material for: DoPI: The Database of Pollinator Interactions
Source: Ecology. 2022 Aug 29;103(11):e3801. doi: 10.1002/ecy.3801 (PMC9786240; doi:10.1002/ecy.3801)
Supplement: Supplementary file 1 — Data S1 [file ECY-103-e3801-s001.zip › MetadataS1.pdf]

DoPI: The Database of Pollinator Interactions

Nicholas J. Balfour[1], Maria Clara Castellanos[1], Dave Goulson[1], Andrew Philippides[2] and Chris Johnson[2]

[1]School of Life Sciences, University of Sussex, Brighton, BN1 9QG, United Kingdom

[2]Department of Engineering and Informatics, University of Sussex, Brighton, BN1 9QG, United Kingdom

Corresponding author: Nicholas J. Balfour; n.balfour@sussex.ac.uk

Introduction

The role of flower-insect pollination interactions in maintaining global biodiversity and agricultural output is well understood, with an estimated 88% of flowering plants and 75% of crops depending on animal pollination to varying degrees (Ollerton et al., 2011, Klein et al., 2007). However, there remain ongoing concerns regarding changes in plant distributions and the long-term decline of both wild and managed insect pollinators (Biesmeijer et al., 2006; Stroh et al., 2014). While several causes have been identified, there is a general consensus that agricultural intensification and habitat loss are key factors (Robinson & Sutherland, 2002; Balfour et al., 2018).

Understanding pollinator-plant interactions is, therefore, vital to optimising: (*i*) conservation strategies for both plants and pollinators (Pocock et al., 2012, Navarro et al., 2017); (*ii*) agricultural pollinator management (Walters et al., 2013; Balfour et al., 2022); (*iii*) research priorities (Vanbergen, 2013); and (*iv*) ecosystem function management (Vamosi et al., 2017). While several studies have collated plant-pollinator networks for analysis (e.g., Welti and Joern, 2015; Mathiasson and Rehan, 2020; Schwarz et al. 2020) there have been few attempts to unite existing datasets and studies into a single depository using a common format (Walters et al., 2013). Recent years have seen notable efforts in this direction (e.g., Brazilian Plant-Pollinator Interactions Network, Saraiva et al., 2017; Plant-pollinator Interactions Database, Redhead et al.

2018; CropPol, Allen-Perkins, 2021), but at present these databases are still in development or lack an interface to explore their contents.

To build on this work, we have created the Database of Pollinator Interactions (DoPI), taking advantage of Great Britain's long and unique history of biological recording. This database primarily collates records of interactions between British insect flower-visitors (bees, wasps, flies, butterflies, moths, beetles, etc.) and their flowering partners (herbs, trees and shrubs). Uniquely, this database aims to provide national-level data at an unprecedented geographical and historical scale. The data include all flower-visiting insects, and for each interaction we rank the evidence for successful pollination. We plan to continue adding records as they are published or unearthed from the literature. Here we provide a description of the development and structure of this dynamic database and accompanying website, and biases inherent in the data.

METADATA

CLASS I – DATASET DESCRIPTORS

A. Data set identity

The Database of Pollinator Interactions

B. Data set and metadata identification code

Data set: DoPI_dataset.csv

C. Data set description

Principal Investigators:

Nicholas J. Balfour, School of Life Sciences, University of Sussex, Brighton, BN1 9QG, United Kingdom

Maria Clara Castellanos, School of Life Sciences, University of Sussex, Brighton, BN1 9QG, United Kingdom

Dave Goulson, School of Life Sciences, University of Sussex, Brighton, BN1 9QG, United Kingdom

Andrew Philippides, Department of Engineering and Informatics, University of Sussex, Brighton, BN1 9QG, United Kingdom

Chris Johnson, Department of Engineering and Informatics, University of Sussex, Brighton, BN1 9QG, United Kingdom

Abstract: Despite the importance of pollinating insects to natural environments and agriculture, there have been few attempts to unite the existing plant-pollinator interaction datasets into a single depository using a common format. Accordingly, we have created one of the world's first online, open-access, and searchable pollinator-plant interaction databases. DoPI (The Database of Pollinator Interactions) was built from a systematic review of the scientific literature and unpublished datasets requested from researchers and organisations. We collated records of interactions between British plant and insect flower-visitor species (or genera), together with associated metadata (date, location, habitat, source publication) where available. The dataset currently (December 2021) contains 101,539 records, detailing over 320,000 interactions. The number of interactions (i.e. the number of times a pairwise species interaction was recorded per occasion) varies considerably among records, averaging 3.6. These include records from 1,888 pollinator species and 1,241 plant species, totalling >17,000 pairwise species interactions. By combining a large volume of information in a single repository, DoPI can be used to answer fundamental ecological questions on the dynamics of pollination interactions in space and time, as well as applied questions in conservation practice. We hope this dynamic database will be a useful tool not only for researchers, but also for conservationists, funding agencies, governmental departments, beekeepers, agronomists and gardeners. We request that this paper is cited when using the data in publications and individual studies where appropriate. Researchers and organisations are encouraged to add further data in the future. The database can be accessed at: https://www.dopi.org.uk/.

D. Keywords:

Bipartite Networks; Conservation; Database; Flower Visitors; Interactions; Phenology; Plants; Pollination; Pollinators.

Description: From the systematic review of the scientific literature and requests to researchers and organisations for unpublished datasets, 302 publications were identified as containing suitable data. The dates of these publications range from 1802 to 2019, but the majority are from this century (95%; 2000-2019). Currently (December 2021), DoPI contains 100,721 records, 88% of which include the number of instances the pairwise interaction was observed (column O, Table 1). The number of interactions varies considerably among records, averaging 3.6. In total DoPI details 329,918 interactions.

The majority of interactions are at the species level, for both plants (80%) and pollinators (92%). These include records from 1,888 pollinator species and 1,241 plant species, totalling >17,000 pairwise species interactions (Fig. 1). The majority of DoPI pollinator records are from bee species (70.6%), followed by flies (18.8%), beetles (6.0%) and butterflies (2.1%). Diptera are the most speciose pollinator order in the database (747 species), followed by Hymenoptera (613), Lepidoptera (240) and Coleoptera (227). *Bombus* (56.3%), *Apis* (10.1%), *Meligethes* (2.7%), *Pegoplata* (2.4%), *Andrena* (1.9%), *Eristalis* (1.9%) and *Episyrphus* (1.9%) are the most frequently recorded pollinator genera. The DoPI plant data is dominated by herbs (70.9%), followed by shrubs (23.7%) and trees (1.6%). Asterales (189 species), Lamiales (156) and Rosales (112) are the most speciose plant orders, with: *Centaurea* (8.0%), *Rubus* (7.9%), *Ranunculus* (5.3%) and *Cirsium* (5.1%) being the most commonly recorded plant genera. The pollinator species recorded visiting the greatest number of plant species were: *Bombus pascuorum* (626), *Apis mellifera* (594), *Bombus terrestris* (590), *Bombus lapidarius* (493) and *Bombus lucorum* (462). The plants species with the greatest number of pollinator species visitors recorded were: *Heracleum sphondylium* (470), *Rubus fruticosus* agg. (349), *Ranunculus repens* (342), *Senecio jacobaea* (308), and *Cirsium arvense* (253). The plant-pollinator networks below (Fig. 1) were generated with all data currently in DoPI, and give a broad overview of the complexity of the British flower visiting communities at the species level. They also offer a visualisation of the contents of the database.

The geographical coverage of the data held by DoPI is skewed towards southern England and central Scotland, with few data from the South and North of Scotland, the Scottish islands and northern England (Fig. 2). Suburban (16.1%), Arable and Horticulture (15.9%), Improved Grassland (14.4%) and Heathland (13.0%) are the most commonly recorded habitats.

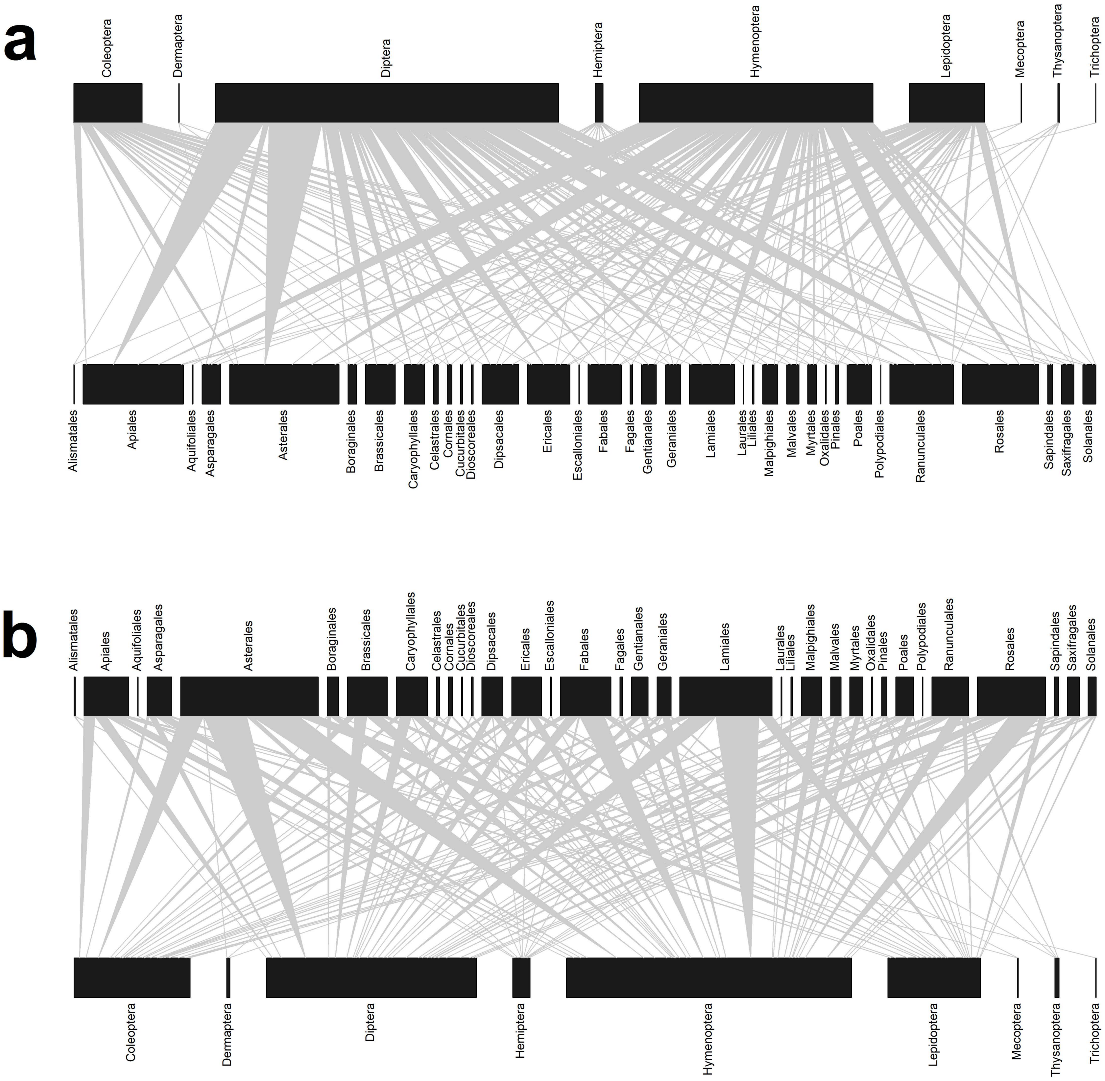

Fig 1. Bipartite networks depicting the number of (a) flower-visitor species (grey lines) recorded visiting each plant order and (b) the number of plant species (grey lines) visited by each flower-visitor order. Insect and plant orders are represented by a rectangle. The width of the rectangles and the width of the lines between them are proportional to the number of (a) insect, or (b) plant, species records.

CLASS II - RESEARCH ORIGIN DESCRIPTORS

A. Overall project description

Identity: A compilation of British plant-pollinator records.

Period of Study: Dates of source publications range from 1802 to 2020.

Objectives: Our study's main goal is to make publicly available unpublished and published records of interactions between British plant and flower-visitor species (or genera), together with associated metadata (date, location, habitat, source publication) where available. Our data set represents one of the first attempts to produce a synthesis of plant-pollinator interactions, with potential applications in community ecology research, agronomy, and establishment of conservation strategies.

Abstract: Same as above.

Sources of funding: The collection of the primary data and compilation of this dataset were funded by grants from the following institutions:

1. Eva Crane Trust (ECTA_20180304)
2. British Beekeepers Association (BBKA_10354)

B. Specific subproject description

Site description: Our dataset is restricted to Great Britain.

Research Methods: We first generated a list of British flower-visitor and plant species, species aggregates, and genera via the National Biodiversity Network (NBN) Atlas (https://nbnatlas.org/), which included: Hymenoptera, Syrphidae, butterflies (all genera described in Tolman, 2008) and all flowering plants. Secondly, a systematic literature review was performed by scraping Google Scholar via the software Publish or Perish (Harzing, 1997) on 24 September 2018. For each pollinator genus (e.g. *Bombus*) we performed a search using the following terms: All of the words: [Bombus], [U.K.]; and, any of the words: [foraging], [forage], [visit], [visiting], [pollinate]. This generated a list of 34,264 publications. Thirdly, these publications were checked, by reading the abstract and results sections, for suitable pollinator-plant interaction records. We also searched the text of volumes 1-20 of *The Entomologist's Record and Journal of Variation* for the word [flower] to identify records. Both direct observations and inferred observations via pollen analysis were added to the database, with the

exception of: (i) birds (class: Aves) and mammals, as no plant species are pollinated by these groups in the UK (Proctor et al., 1996), (ii) insects assumed to be feeding on the plant's phloem sap, rather than nectar or pollen, e.g., aphids (family: Aphididae), (iii) plants or pollinators that could not be associated with either a genus, species or species aggregate listed in the NBN Atlas (https://nbnatlas.org/). When encountering species records that did not match those listed in the NBN Atlas we searched the Global Biodiversity Information Facility (https://www.gbif.org/) for synonyms.

For each record we collated the following metadata, where available: plant and flower-visitor species/genus NBN code, Catalogue of Life (COL) code, date, location, habitat, methodology (Table 1). We classified each record in terms of the strength of evidence that the floral visitor was an effective pollinator, using the scale detailed in Column X (Table 1). We also recorded whether the visitor was reported to be collecting nectar or pollen. Each record in the database is associated with a source publication (author(s), year, journal details, DOI, URL), allowing users to refer to the original literature/author for further information, if required. Errors may be present in the database due to mistakes in the original literature, interpretation of the data, or data entry. Extensive attempts have been made to minimize errors, especially of the third kind. This was primarily achieved by summarising and visualising the data, and by checking for consistency between pollinator and plant species names (Columns K and N, Table 1) and their NBN codes (Columns J and M, Table 1), as well as between the source publication's PaperID (unique number assigned to each publication) and its DOI (Column F, Table 1). We encourage users to report any errors encountered in the database.

Table 1. Details of output in comma-separated values (CSV) files available from the Database of Pollinator Interactions. Data are given in 27 columns (Columns A-AB) with headers (Titles) and accompanying notes.

Column	Title	Notes
A	Authors	Publication author(s)
B	Title	Publication title
C	Journal	Publication journal
D	Pub Year	Publication year
E	Pub Vol	Publication volume
F	DOI	Publication Digital Object Identifier (https://www.doi.org)
G	Methodology	Methodology used in data collection.
H	Pollinator Survey	Survey effort in data collection (All, Group, Single Species)
I	Plant Survey	Survey effort in data collection (All, Group, Single Species)
J	NBN Pollinator Code	Pollinator species code from the NBN Atlas (https://nbnatlas.org/)
K	COL Pollinator Code	Pollinator species code from the Catalogue of Life (https://www.catalogueoflife.org/)
L	Pollinator Species	Pollinator species name from the NBN Atlas
M	Caste	Caste or gender of pollinator
N	NBN Plant Code	Plant species code from the NBN Atlas
O	COL Plant Code	Plant species code from the Catalogue of Life (https://www.catalogueoflife.org/)
P	Plant Species	Plant species name from the NBN Atlas
Q	Interactions	Number of interactions recorded
R	Date	Day of the month of interaction
S	Month	Month of interaction
T	Year	Year of interaction
U	Grid Letter	Location of interaction. Letters from the Ordnance Survey National Grid
V	Grid Code	Location of interaction. Numbers from the Ordnance Survey National Grid
W	Latitude	Location of interaction given in geographic coordinates
X	Longitude	Location of interaction given in geographic coordinates
Y	Habitat	Habitat type in which the interaction was recorded. Loosely based on those used on the Centre of Ecology and Hydrology Land Cover Map 2007 (https://www.ceh.ac.uk/services/land-cover-map-2007)
Z	Pollination	Pollination quality of interaction, i.e. effectiveness of visitor to pollinate the flower. Based on Ollerton et al. (2019) and Adams & Lawson (1993):<br>1 - pollination confirmed, visitors with pollen attached and observed to produce pollination of a flower (e.g., transferring pollen to stigmas and/or leading to seed set)<br>2 - pollination inferred, visitors observed with pollen attached (but not confirmed to transfer pollen to stigmas)<br>3 - pollination inferred from circumstantial evidence (e.g., visitors observed on flowers, but evidence of picking up pollen is missing)<br>4 - no pollination, the flower-visitor is a nectar or pollen robber, a herbivore, a predator, or a parasite of insects in the flowers
AA	Pollen	Pollen collection by pollinator species reported
AB	Nectar	Nectar collection by pollinator species reported
AC	Record	Record unique reference number
AD	ArticleURL	Publication web address

The DoPI online interface was created using the open-source software tools, MySQL (version 5.6, http://www.mysql.com/) and PHP (version 5.6, http://www.php.net/). MySQL is a relational database system ideally suited for rapid retrieval of relatively static records. The data are currently stored in four linked Excel tables: (i) one detailing individual interactions and their metadata, (ii, iii) two listing UK (native and exotic) plant and pollinator species and genera retrieved from the NBN Atlas, their taxonomic ranks), NBN Atlas codes (https://nbnatlas.org/), and COL codes (https://www.catalogueoflife.org/) and (iv) one detailing the list of publications retrieved by the systematic review. Users may search and filter the stored data via the online interface using five categories: (i) pollinators (groups, common names or taxonomic rank), (ii) plants (groups, common names or taxonomic rank), (iii) habitat type, (iv) location, and (v) date (years, months, and months in years). The data can be viewed on the website and downloaded as comma-separated values (CSV) files for analysis. The format of the CSV files is outlined in Table 1. The dataset associated with this publication also follows this format.

Database Validations: We ran a series of validations to detect potential taxonomic, geographical and other biases in DoPI. This was achieved by assessing the number of records across different taxonomic groups, years and habitat types. To provide a geographical overview, we mapped the interactions across Britain using the R package "ggplot2" (Wickham, 2011). To ascertain whether the database currently captures both specialised and generalist pollination interactions, we used the list of British specialist vs. generalist plants identified by Maia (2019) using centrality metrics (González, et al., 2010). We compared the number of pollinator species recorded visiting, and the number of publications associated, with the 15 plant species with the highest centrality score with the 15 with the lowest score. These data were analysed via ANOVA (R *aov* function).

The data stored in DoPI are broadly reflective of those available from previous research and the efforts of British recording groups and are therefore not without biases. For instance, the majority of records are from southern England and central Scotland, likely reflecting the distribution of the British populace. Furthermore, over a quarter of the observations have been recorded in urban or suburban habitats (25.6%), which are estimated to occupy only 7.5% of the UK (Morton et al., 2011). Likewise, heathland is overrepresented (13.0% vs 6.0%; Morton et al., 2011). The data also show a preponderance of records from bee species (70.6%), and a low

number from beetle and moth species. This could be partly explained by a sampling bias toward daytime observations (Bernhardt, 2000; Devoto et al., 2011). In addition, there is a low number of records from tree species, possibly due to biases towards sampling during summer (since the majority of trees flower in spring; Balfour et al., 2018), and/or the practical difficulties of recording visits to tall plants, as well as the exclusion of trees from most pollinator surveys (Rotheray and Gilbert, 2011; Balfour et al., 2015). Finally, twice the number of pollinator species were recorded visiting the generalist vs. specialist plant species (101.7 vs. 50.0) identified by Maia (2019). However, the number of publications with records of generalist vs. specialist plant species did not differ significantly (15.6 vs. 16.3; ANOVA, $F_{1,28} = 0.03$, $P = 0.874$). Whilst this analysis indicates that DoPI captures both specialized and generalist pollination interactions, it is likely that many specialist interactions are missing from the database. Users are urged to account for these biases in any analysis of the database, particularly regarding higher-level analyses (e.g., network metrics).

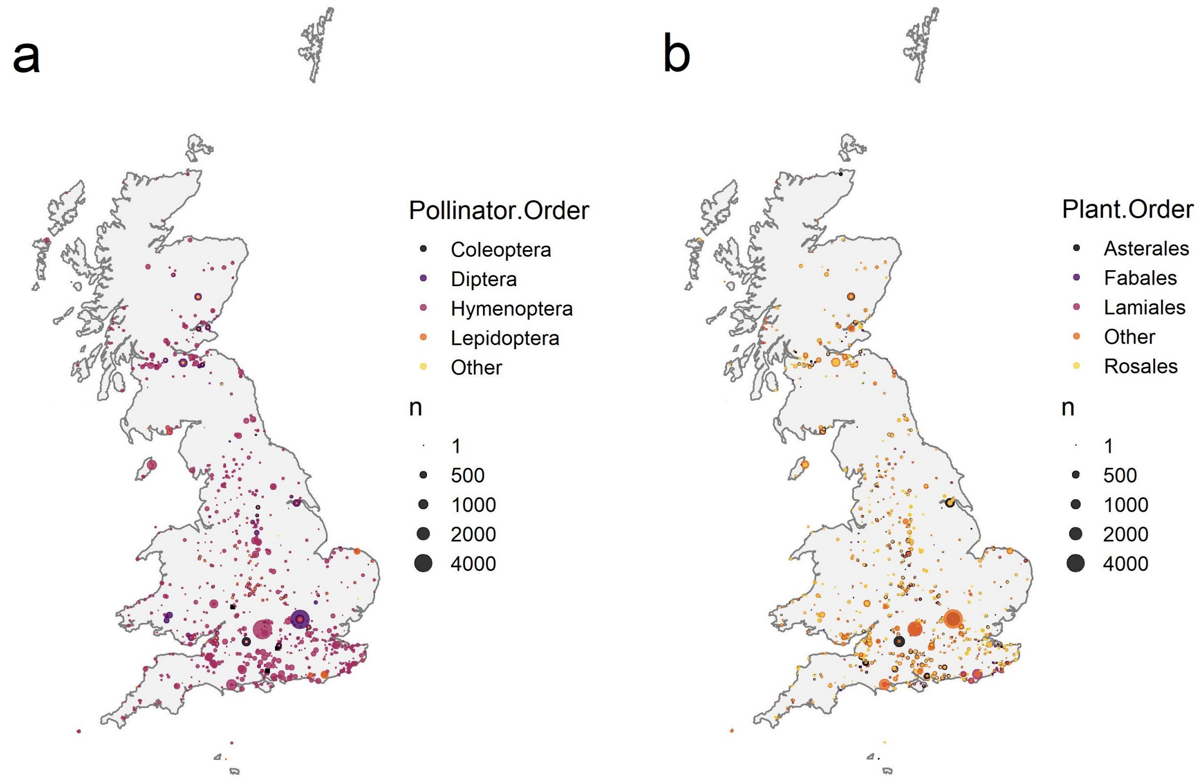

Fig. 2 Maps of the geographical coverage of the (a) pollinator and (b) plant records held by the Database of Pollinator Interactions. The size of the circles represents the number of records from each location.

This database could readily be extended to include suitably curated publicly generated records in the future, thereby increasing the value of the growing number of citizen science projects that are producing large volumes of data. So far, DoPI is focused on Britain, but the software and methodology could readily be applied to data from other countries, and we welcome collaborations with international groups pursuing similar goals. To allow such integration with future international datasets, the PHP and MySQL code underlying our database and accompanying supporting documents are freely available by request from the lead author.

CLASS III - DATA SET STATUS AND ACCESSIBILITY

A. Status

Latest update: December 2021

Latest Archive date: December 2021

Metadata Status: Latest update December 2021, version submitted

Data verification: Data from published and unpublished sources. We searched for extreme values, corrected any transcription errors, and homogenised text and the taxonomic information. Special characters were removed from the data set.

B. Accessibility

Storage location and medium: The data set can be accessed as Supporting Information to this publication in *Ecology* in .csv format as well on the University of Sussex, Life Sciences website (https://www.sussex.ac.uk/lifesci/ebe/dopi) and at https://www.dopi.org.uk/.

Contact persons: Nicholas J. Balfour, School of Life Sciences, University of Sussex, Brighton, BN1 9QG, United Kingdom. Email: n.balfour@sussex.ac.uk

Copyright restrictions: None

Proprietary restrictions: Please cite this data paper when the data are used in publications. We also request that researchers and teachers let us know how they are using the data.

Costs: None

CLASS IV - DATA STRUCTURAL DESCRIPTORS

A. Data set File

Identity: DoPI_dataset.csv

Size: 101,539 records, 33.8 MB

Format and storage mode: Comma separate value format (csv)

Header Information: See column descriptions in Table 1.

Alphanumeric attributes: Mixed.

Data anomalies: If no information is available for a given record, this is indicated as 'NA'.

Data reading suggestion in R: read.csv ("DoPI_dataset.csv", header = TRUE)

B. Variable Information

Table 1. Description of the fields related to the data set linked to the file DoPI_dataset.csv.

CLASS V - SUPPLEMENTAL DESCRIPTORS

A. Data acquisition:

Location of completed data forms: The data are saved in computers maintained by Nicholas J. Balfour (see above for institutional address).

Data entry verification procedures: Data were digitized by technicians inputting the data into Excel spreadsheets. Nicholas J. Balfour reviewed and compiled the data into the master database.

B. Quality assurance/quality control procedures:

The database was extensively reviewed, validated, and curated between 2018-2021. Data validation was achieved by summarising and visualising the data, searching for outliers, checking for consistency between pollinator and plant species names (Columns K and N, Table 1) and their NBN codes (Columns J and M, Table 1), as well as between the source publication's PaperID (unique number assigned to each publication) and its DOI (Column F, Table 1).

C. Computer programs and data-processing algorithm:

The DoPI online interface was created using the open-source software tools, MySQL (version 5.6, http://www.mysql.com/) and PHP (version 5.6, http://www.php.net/).

D. Archiving:

The dataset is archived with this *Ecology* paper.

Acknowledgements

We thank our funders, the British Beekeepers Association and the Eva Crane Trust, three anonymous reviewers for their thoughtful and thorough comments, Tom Dando, Sam Gandy, Maria Chiara Perusini, Rosalind Henderson, Shannon Phillips, Tomasz Kowalcyk, Ciaran Harris, Cassandra Uthoff, Natalie Cook, Anina Day, Tom Green, Paul Silver, and the researchers and organisations who have kindly contributed their data.

References

Adams, P.B., and Lawson, S.D. 1993. Pollination in Australian orchids: a critical-assessment of the literature 1882-1992. Australian Journal of Botany 41:553-575.

Allen-Perkins, A., Magrach, A., Dainese, M., Garibaldi, L.A., Kleijn, D., Rader, R., Reilly, J.R., Winfree, R., Lundin, O., McGrady, C.M., and Brittain, C. 2022. CropPol: a dynamic, open and global database on crop pollination. Ecology 103:e3614.

Balfour, N.J., Fensome, K.A., Samuelson, E.E., and Ratnieks, F.L. 2015. Following the dance: ground survey of flowers and flower-visiting insects in a summer foraging hotspot identified via honey bee waggle dance decoding. Agriculture, Ecosystems & Environment 213:265-271.

Balfour, N.J., Ollerton, J., Castellanos, M.C., and Ratnieks, F.L. 2018. British phenological records indicate high diversity and extinction rates among late-summer-flying pollinators. Biological Conservation 222:278-283.

Balfour, N.J., and Ratnieks, F.L. 2022. The disproportionate value of 'weeds' to pollinators and biodiversity. Journal of Applied Ecology 59:1209-1218.

Bernhardt, P., 2000. Convergent evolution and adaptive radiation of beetle-pollinated angiosperms. Plant Systematics and Evolution 222:293–320

Biesmeijer, J.C., Roberts, S.P., Reemer, M., Ohlemuller, R., Edwards, M., Peeters, T., Schaffers, A.P., Potts, S.G., Kleukers, R.J.M.C., Thomas, C.D., and Settele, J. 2006. Parallel declines in pollinators and insect-pollinated plants in Britain and the Netherlands. Science 313:351-354.

Devoto M., Bailey S., and Memmott J. 2011. The 'night shift': Nocturnal pollen-transport networks in a boreal pine forest. Ecological Entomology 36:25–35.

González, A.M.M., Dalsgaard, B., and Olesen, J.M. 2010. Centrality measures and the importance of generalist species in pollination networks. Ecological Complexity 7:36-43.

Harzing, A., 1997. Publish or perish. https://harzing.com/resources/publish-or-perish. Tarma Software Research Pty Limited.

Klein, A.M., Vaissiere, B.E., Cane, J.H., Steffan-Dewenter, I., Cunningham, S.A., Kremen, C., and Tscharntke T. 2007. Importance of pollinators in changing landscapes for world crops. Proceedings of the National Academy of Sciences 274:303-313.

Maia, K.P. 2019. Bottom-up effects in plant-insect networks: the role of plant communities in structuring insect communities (Doctoral dissertation, University of Bristol).

Mathiasson, M.E., and Rehan, S.M. 2020. Wild bee declines linked to plant-pollinator network changes and plant species introductions. Insect Conservation and Diversity 13:595-605.

Morton, D., Rowland, C., Wood, C., Meek, L., Marston, C., Smith, G., Wadsworth, and R., Simpson, I.C. 2011. Final Report for LCM2007 – The New UK Land Cover Map. CS Technical, Report No 11/07.

Navarro, L.M., Fernández, N., Guerra, C., Guralnick, R., Kissling, W.D., Londoño, M.C., Muller-Karger, F., Turak, E., Balvanera, P., Costello, M.J., and Delavaud, A. 2017. Monitoring biodiversity change through effective global coordination. Current Opinion in Environmental Sustainability, 29:158-169.

Ollerton, J., Winfree, R., and Tarrant, S. 2011. How many flowering plants are pollinated by animals? Oikos 120:321-326.

Ollerton, J., Liede-Schumann, S., Endress, M.E., Meve, U., Rech, A.R., Shuttleworth, A., Keller, H.A., Fishbein, M., Alvarado-Cárdenas, L.O., Amorim, F.W., and Bernhardt, P. 2019. The diversity and evolution of pollination systems in large plant clades: Apocynaceae as a case study. Annals of Botany 123:311-325.

Pocock, M.J., Evans, D.M., and Memmott, J. 2012. The robustness and restoration of a network of ecological networks. Science 335:973-977.

Proctor, M., Yeo, P., and Lack, A. 1996. *The natural history of pollination*. HarperCollins Publishers, New York.

R-Project. 2021. R: A language and environment for statistical computing. R Foundation, Vienna, Austria. URL http://www.r-project.org/. Accessed 10 January 2021.

Redhead, J.W., Woodcock, B.A., Pocock, M.J., Pywell, R.F., Vanbergen, A.J., and Oliver, T.H., 2018. Potential landscape-scale pollinator networks across Great Britain: structure, stability and influence of agricultural land cover. Ecology Letters 21:1821-1832.

Robinson, R.A., and Sutherland, W.J. 2002. Post-war changes in arable farming and biodiversity in Great Britain. Journal of Applied Ecology 39:157-176.

Rotheray, G.E., and Gilbert, F. 2011. *The natural history of hoverflies*. Forrest Text, Ceredigion, UK.

Saraiva, A., Salim, J., Agostini, K., Wolowski, M., Silva, J., Veiga, A., and Albertini, B. 2017. Brazilian Plant-Pollinator Interactions Network: definition of a data standard for digitization, sharing, and aggregation of plant-pollinator interaction data. Biodiversity Information Science and Standards, 1:e20298.

Schwarz, B., Vázquez, D.P., CaraDonna, P.J., Knight, T.M., Benadi, G., Dormann, C.F., Gauzens, B., Motivans, E., Resasco, J., Blüthgen, N., and Burkle, L.A. 2020. Temporal scale-dependence of plant–pollinator networks. Oikos 129:1289-1302.

Stroh, P.A., et al. 2014. *A Vascular Plant Red List for England*. Botanical Society of Britain and Ireland, Bristol.

Tolman, T. 2008. *Collins butterfly guide*. Harper Collins, London, UK.

Vamosi, J.C., Gong, Y.B., Adamowicz, S.J., and Packer, L. 2017. Forecasting pollination declines through DNA barcoding: the potential contributions of macroecological and macroevolutionary scales of inquiry. New Phytologist 214:11-18.

Vanbergen, A.J. 2013. Threats to an ecosystem service: pressures on pollinators. *Frontiers in* Ecology and the Environment 11:251-259.

Walters, M., Pereira, H.M., Ferrier, S., Geller, G.N., Jongman, R., Scholes, R.J., Bruford, M., and Reyers, B. 2013. *Essential Biodiversity Variables*. In Convention on Biological Diversity, Subsidiary Body on Scientific, Technical, and Technological Advice.

Welti, E.A., and Joern, A. 2015. Structure of trophic and mutualistic networks across broad environmental gradients. Ecology and Evolution 5 326-334.

Wickham, H. 2011. ggplot2. Wiley Interdisciplinary Reviews: Computational Statistics, 3, 180-185.
